# First Description of the Breeding Biology of the Spectacled Fulvetta (*Fulvetta ruficapilla sordidior*) in Southwest China

**DOI:** 10.3390/ani13132157

**Published:** 2023-06-30

**Authors:** Shixiang Fan, Jiansong Zhang, Yubao Duan, Xu Luo

**Affiliations:** 1 Key Laboratory for Forest Resources Conservation and Utilization in the Southwest Mountains of China, Ministry of Education, Faculty of Biodiversity and Conservation, Southwest Forestry University, Kunming 650224, China; shixiang_fan@163.com (S.F.);; 2 Asian Elephant Research Center of National Forestry and Grassland Administration, Southwest Survey and Planning Institute of National Forestry and Grassland Administration, Kunming 650216, China

**Keywords:** breeding biology, life history traits, *Fulvetta ruficapilla sordidior*

## Abstract

**Simple Summary:**

The Spectacled Fulvetta (*Fulvetta ruficapilla sordidior*) is an endemic bird species of the mountains in southwest China. The breeding biology of this species is largely unknown. In order to describe the breeding biology of this species, this study was conducted for two years on Humashan Mountain, a hill mainly covered with secondary forest located at the eastern marginal of Kunming, the capital city of Yunnan Province in Southwest China. A total of 16 nests were found, mainly located in dense shrubs. The breeding season is from March to June, with its peak period from April to May. The clutch size is 2–3, most of which are 3 eggs. The incubation period is 13–14 days, and the nesting period is 12–14 days. Both parents participated in the breeding process. We found a total of 16 nests, of which only 11 were active; 5 were successfully fledged with at least one (45.45%). This study is the first report on the breeding biology of the Spectacled Fulvetta, which has scientific value for the understanding of this species and other relative species in the *Fulvetta* genus.

**Abstract:**

The Spectacled Fulvetta (*Fulvetta ruficapilla sordidior*) is an endemic bird species to the southwest mountains of China, distributing from 1250 to 2500 m in the widespread broadleaved evergreen forest and occasionally in secondary scrubs. The present study describes its breeding biology for the first time. Fieldwork was conducted in the springs of 2017 and 2018 on Humashan Mountain, a hill mainly covered by secondary forest located at the eastern marginal of Kunming, the capital of Yunnan Province in Southwest China. This bird was found to initiate egg-laying mainly in March, and most nestlings fledged in late April and May. There were 16 nests found in total, which were located mainly in the dense shrubs at a height of 0.99 ± 0.40 m (*n* = 15). Of the 11 active nests, clutch size averaged 2.73 ± 0.45 (*n* = 11). Focal observations were made on nests; the incubation lasted for 13.67 ± 0.47 days (*n* = 3) with a notably high nest attendance, i.e., eggs were incubated 84.23% of the observation time. Nestlings fledged at 13.00 ± 0.71 days (*n* = 4), and parents feeding frequency increased as the nestlings grew. Overall, the cumulative hatching and fledgling rates were 71.43% and 35.71%, resulting in a nesting success rate of 45.45%.

## 1. Introduction

While avian breeding biology is critical for the understanding of life history theory and how bird species respond to climate and land use changes, only one third of the extant birds for which breeding biology has been well documented [1,2,3,4,5]. This echoes the problem that the lack of life history research for a great number of species remains one of the largest challenges for ornithologists [2]. With respect to habitat types and regions, forest birds in Asia are among the least studied groups [5]. The babblers are a diverse group of passerine birds, with more than 450 species distributed worldwide and more than 170 species in China [6,7]. They are one of the groups lacking breeding biology data [5].

The *Fulvetta* genus is a poorly studied group of understory forest babblers. This genus currently comprises eight species, including Spectacled Fulvetta (*Fulvetta ruficapilla*), Chinese Fulvetta (*F. striaticollis*), Brown-throated Fulvetta (*F. ludlowi*), Indochinese Fulvetta (*F. danisi*), White-browed Fulvetta (*F. vinipectus*), Manipur Fulvetta (*F. manipurensis*), Gray-hooded Fulvetta (*F. cinereiceps*), and Taiwan Fulvetta (*F. formosana*). They distribute west from the Himalayan Mountains to the east edge of the Asian continent and islands [6]. The eight species in this genus are all resident birds in China, and they move around in a small range all year round. They are relatively small in size, with a body length of about 12 cm and a weight of about 10 g [6,8]. Their distribution spans a wide range of elevations, from lowlands to plateaus [8]. To date, the detailed breeding biology has only been studied for White-browed Fulvetta (*F. vinipectus*) [9] and Gray-hooded Fulvetta (*F. cinereiceps*) [10].

Spectacled Fulvetta is a small understory bird that dwells in evergreen forest and dense secondary scrubs at elevations of 1250–2500 m, found only in the southwest and central mountainous areas of China [11]. It comprises two subspecies, including *F. r. ruficapilla* in Central China and *F. r. sordidior* in Southwest China [6]. Although it is listed as “least concern” by the International Union for Conservation of Nature (IUCN) Red List, which indicates the population trend is increasing [12], the lack of understanding of its breeding biology is a fact that is not conducive to the evaluation and protection of this species in the future. No breeding information on the Spectacled Fulvetta was available until Li et al. [13], who briefly noted two nests and the breeding habitats of *F. r. ruficapilla* in the Qinling Mountains in Central China. Yet, the critical breeding parameters of this species were not documented.

Here, we provide the first detailed information on the breeding biology of the *F. r. sordidior*, including the nest, eggs, clutch size, incubation period, nestling period, nestlings, nesting success, and parental care behavior in Yunnan Province, Southwest China. We also compare the breeding parameters of the *F. r. sordidor* with those of the White-browed Fulvetta [9] and the Gray-hooded Fulvetta [10].

## 2. Materials and Methods

### 2.1. Study Site

**Figure 1:**
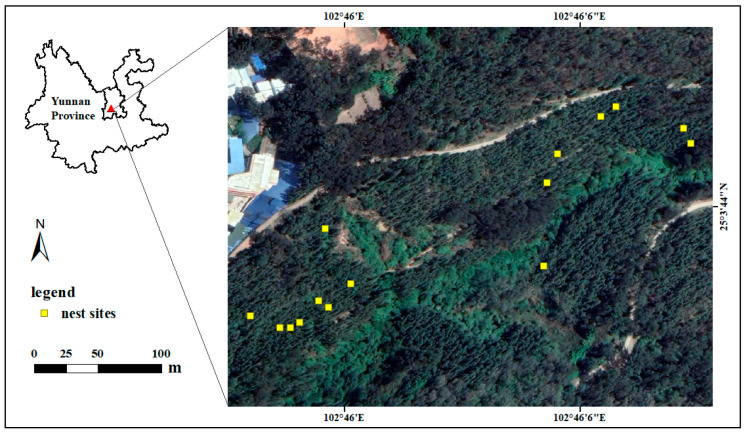
Study area and nest sites of the Spectacled Fulvetta, Yunnan, China.

The field work was conducted at Humashan Mountain (25°03′ N, 102°46′ E), a hill from 1918 to 2106 m, located east of Kunming city, Yunnan Province, Southwest China (Figure 1). Although this area is adjacent to the urbanized city area, secondary forests form the dominant vegetation, and some patches of primary broadleaved-evergreen forests remain in valleys. Trees include pine (*Pinus yunnanensis*), eucalyptus (*Eucalyptus globules*), and cypress (*Platycladus orientalis*). Shrubs include osyris (*Osyris wightiana*), myrsine (*Myrsine africana*), and camellia (*Camellia japonica*). Herbals include arundinella (*Arundinella anomala*), crofton weed (*Ageratina adenophora*), and sticktight (*Bidens bipinnata*) [14]. The secondary-growth scrubs and herbs have become very dense since logging was restricted by the local forestry administrations. The climate is typical temperate, with a mean annual temperature of 16.5 °C [15]. The precipitation varies between the wet season (May–September) and the dry season (October–next April), with a mean annual precipitation of ~1450 mm [15].

### 2.2. Field Procedures and Data Analysis

Our fieldwork was conducted from March to June in both 2017 and 2018. We observe the clustering and activity range of the Spectacled Fulvetta during non-breeding and breeding seasons to enable us to determine the changes in population size and distribution range before and after breeding. We began searching for breeding pairs or nests on Humashan Mountain in early March. The field survey was conducted along the existing trails or roads. When individual pairs were seen copulating or carrying nest materials, we followed the individual until the nest was found or marked the locations for the next search attempt [16]. The coordinates of each nest were recorded with a GPS recorder (Garmin Co., Ltd., Taibei City, China), and the found nests were marked. To minimize the researcher’s disturbance of breeding activities, we then visited the nests every two–three days to check the breeding status of the paired individuals. At each visit to each nest, we recorded the stages (i.e., nest construction, incubation, and nestling) and fate (i.e., success or failure) of the breeding pairs. In the incubation and nesting periods, we also noted the number of eggs and nestlings in each nest.

We defined the incubation period as the time from the commencement of incubation to the hatching of the chicks, and the nestling period as the time between the hatching date and the fledging date [17]. When egg incubation and nestling feeding began, we observed incubation and feeding behaviors using binoculars (10 × 42, Shuntu Optical Co., Ltd., Kunming, China). To reduce the disturbance to the breeding birds, observers stayed at a concealed location that was more than 5 m away from the nest. To increase monitoring efforts and record potential predators, we installed digital cameras (Hero 5, GoPro, San Mateo, CA, USA) to videotape some nests. The digital cameras were set up or replaced only when the parent bird left the nest. Each observation and videotaping last for at least one hour. For these observations, we recorded the duration of incubation and brooding and the time of provisioning events.

We measured the size of the eggs, including egg length, egg width, and egg mass. We measured the morphological traits of five nestlings in three nests. These included body mass, body length, bill length, wing length, tarsus length, and tail length. To reduce anthropogenic disturbance, we measured eggs and nestlings only when the parent birds were absent. We measured nests and examined the nest materials after the nestlings fledged or breeding failed. Specifically, we measured nest height, nest depth, inter-diameter, outer diameter, and from ground level to the bottom of the bird’s nest, using a sliding caliper measuring to the nearest 1.0 mm [18].

Finally, we calculated the breeding success of the Spectacled Fulvetta. We divided the total number of hatchlings and fledglings by the total number of laid eggs to calculate hatching and fledging success, respectively [19]. We considered a nest successful if it fledged at least one young [20].

We used the Linear Mixed Effects Models to assess how feeding behavior, as indicated by the proportion of brooding time and feeding frequency, changes through the age of the nestlings. In each model, the proportion of brooding time and feeding frequency were the response variables, respectively. In each model, the age of nestlings is the predictor, and the nest ID is modeled as the random effect. RStudio (10 March 2022, RStudio, PBC, Vienna, Austria) and R package nlme were used for statistical analyses [21,22], and data are presented as mean ± SD.

## 3. Results

### 3.1. Breeding Phenology

**Figure 2:**
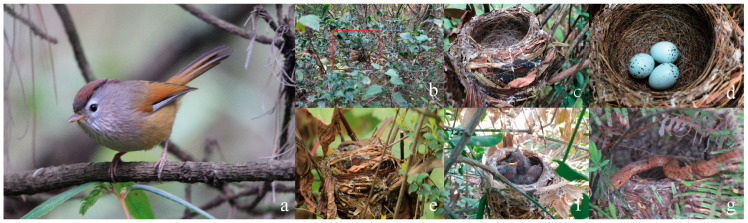
The adult, habitat, nest, eggs, nestlings, and predator of the Spectacled Fulvetta. (**a**) Adult; (**b**) nest’s surroundings; (**c**) nest; (**d**) eggs; (**e**) incubating bird; (**f**) nestlings before fledging; and (**g**) nestling predator King Ratsnake (*Elaphe carinata*). The photos were taken by the authors.

The Spectacled Fulvetta formed small groups during non-breeding seasons, while they were divided into breeding pairs in late February or early March. In the field, it is difficult to distinguish the male from the female (Figure 2a). In both 2017 and 2018, the Spectacled Fulvetta initiated breeding in early March and completed incubation and nesting in late June. We observed the earliest nest-material-carrying attempt on 8 March 2017, soon after the formation of breeding pairs. Egg-laying was initiated in mid-March, and the mean initiation dates were 19 April (in 2017) (*n* = 6) and 11 April (2018) (*n* = 3), respectively. In 2017, the earliest nest was found on 25 March, with three eggs in the nest. In 2018, the earliest nest was found on 25 March too, but it was still in the nest construction period. The latest fledglings in these nests were observed on 30 June 2017, and 11 May 2018, respectively.

### 3.2. Nest and Nest Sites

We found a total of 16 nests (nine in 2017 and seven in 2018), of which only 11 were active. Among these active nests, one (9.10%) was found during nest-building, eight nests (72.72%) during incubation, and two (18.18%) during the nestling period. Nests were found in the undergrowth bushes of eucalyptus (37.5%, *n* = 6) or cypress trees (62.5 = %, *n* = 10), at a height of 0.99 ± 0.40 m (range = 0.42–2.10 m, *n* = 15) above the ground (Figure 2b).

The nest was cup-shaped and was made primarily of dry leaves, grass, and fibers from wood skin, and the inner lining was filled with soft fibers, mammalian hair, and some plastic filaments (Figure 2c). Arthropod silk was found on the surface or around the cup openings in all nests. The nest height and outer nest diameter were 64.35 ± 8.11 mm (range = 53.99–83.82 mm) and 71.34 ± 3.28 mm (range = 65.82–77.82 mm), respectively (*n* = 15). The depth and inner diameter of the nests were 45.99 ± 4.15 mm (range = 40.20–51.88 mm) and 48.88 ± 3.34 mm (range = 43.32–55.28 mm), respectively (*n* = 15).

### 3.3. Eggs and Incubation

There were two or three eggs in the nests, resulting in a mean clutch size of 2.73 ± 0.45 (*n* = 11). Eggs were ovoid with white coloration and covered at the blunt end by dark blotches of varied size (Figure 2d). The dimensions of the five measured eggs were 17.21 ± 0.43 mm (range = 16.67–17.78 mm) in length and 13.02 ± 0.48 mm (range = 12.45–13.78 mm) in width. Fresh egg mass was 1.28 ± 0.13 g (range = 1.13–1.50 g, *n* = 5 eggs in 4 nests).

The Spectacled Fulvetta laid one egg each day, and the incubation started after the clutch was completed (Figure 2e). During the incubation period, we focused our observations on six nests. Each observation lasted for at least one hour, totaling 1998 min in 14 days (9 d in 2017 and 5 d in 2018), while 84.23% of the observed time was a bird incubating. The incubation period lasted for 13.67 ± 0.47 days (range = 13–14 d, *n* = 3). We observed both parents taking turns incubating. On average, the incubating bout duration was 27.56 ± 12.67 min (range = 5–62 min, *n* = 50). The time without the presence of parent birds (off-bunt duration) in the nest was 7.56 ± 6.99 min (range = 1–28 min, *n* = 50).

### 3.4. Nestlings

**Figure 3:**
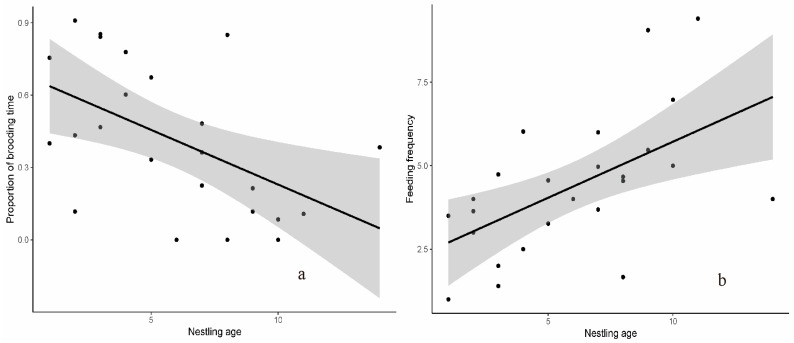
The fluctuation of the proportion of brooding time and feeding frequency of the Spectacled Fulvetta. (**a**) Proportion of brooding time (*p* < 0.05); (**b**) feeding frequency (*p* < 0.01).

The paired birds were observed taking turns brooding nestlings, feeding nestlings, and removing fecal sacs during the nestling period. During the nestling period, by direct observations seven nests were monitored for 26 days (16 days in 5 nests in 2017 and 10 days in 2 nests in 2018), totaling 3518 min. In total, 45.38% of the observed period was when a bird was brooding nestlings. We recorded 67 events of nestling brooding behavior. The duration time of each brooding was 15.52 ± 9.00 min (range = 2–27 min), which mostly occurred in the first 10 days and seldom happened after 10 days (Figure 3a). We recorded the parents feeding nestlings in 241 events that averaged 9.41 times per hour, and the feeding interval was 12.99 ±9.48 min (range = 2–45 min). We found that feeding frequency increased as the nestlings grew and feeding intervals became shorter (Figure 3b). We recorded 47 events related to nest-cleaning behavior, of which 25 were parents eating fecal sacs directly in the nest and 22 were parents taking fecal sacs out of the nest.

Nestlings on the first day were naked with their eyes closed. On the 7th day, nestlings began to develop tail feathers. Nestlings on the 11th day were similar in plumage morphology to adults but with a distinctive yellow bill (Figure 2f). Nestlings left the nest 13.00 ± 0.71 days after being hatched (range = 12–14 d, *n* = 4). New nestlings weighed 2.33 ± 0.11 g (range = 2.19–2.42 g, *n* = 5), had a body length of 29.80 ± 0.80 mm (range = 28.62–30.68 mm), a wing length of 8.26 ± 1.20 mm (range = 6.64–10.02 mm), a tarsus length of 8.42 ± 0.62 mm (range = 7.56–9.48 mm), and a bill length of 3.73 ± 0.42 mm (range = 3.28–4.48 mm). Near-fledglings weighted 9.43 ± 0.88 g (range = 8.33–10.58 g, *n* = 5), with body length of 55.62 ± 4.15 mm (range = 49.58–61.02 mm), wing length of 35.38 ± 3.56 mm (range = 29.26–39.26 mm), tarsus length of 24.22 ± 2.23 mm (range = 22.26–27.32 mm), bill length of 7.02 ± 0.81 mm (range = 6.25–8.32 mm), and tail length of 6.3 ± 0.58 mm (range = 5.82–7.12 mm).

### 3.5. Nesting Success and Predation

Of the 11 active nests, three (27.27%) failed after egg-laying, three (27.27%) failed during nestling periods, and five (45.45%) successfully fledged with at least one fledgling. The cumulative hatching and fledging rates were 71.43% and 35.71%, corresponding to 20 hatchlings and 10 fledglings from the 28 laid eggs, respectively. The average number of fledglings per successful nest was 2.00 ± 0.89 (range = 1–3, *n* = 5). The overall nesting success of 11 nests was 45.45%. In one of the failed nests, three nestlings were depredated by a juvenile King Ratsnake *(Elaphe carinata*) (Figure 2g). The other five nests failed for unknown reasons.

## 4. Discussion

In the present study, we provide the first detailed information about the breeding biology of the Spectacled Fulvetta in Southwest China. This species was found to initiate egg-laying mainly in March, and most nestlings fledged in late April and May. The nests were located in dense bushes. The clutch size was 2–3 (*n* = 11), most of which were 3 eggs (54.55%). The incubation period was 13–14 days (*n* = 3), and the nesting period was 12–14 days (*n* = 4). The nest material consisted of tree bark, withered stems, hair, thin plastic threads, and plastic sheets. The nest was bowl-shaped and was opening up. The nest was 0.99 ± 0.40 m (*n* = 15) above the ground. Eggs were ovoid with white coloration and covered at the blunt end by dark blotches of varied size. The dimensions of five eggs were 17.21 ± 0.43 mm (*n* = 5) in length and 13.02 ± 0.48 mm (*n* = 5) in width. Fresh egg mass was 1.28 ± 0.13 g (*n* = 5). The cumulative hatching and fledging rates were 71.43% and 35.71%, respectively. The breeding success of 11 nests was 45.45%.

Comparing our findings with the documentation of two nests of *F. r. ruficapilla* in Shaanxi Province, the clutch size in our study is relatively smaller (Table 1). A previous study found that the clutch size of passerine birds tended to increase with an increase in latitude [23]. Despite the low sample sizes, our findings were consistent with this pattern. We also compared our findings with the existing breeding information of the other two fulvettas, the White-browed Fulvetta [9] and the Gray-hooded Fulvetta [10]. All three species preferred dense bushes and shrubs as the nesting substrate. Dense bushes and shrubs could provide excellent cover for their nests and offspring. With respect to breeding altitudes, the Spectacled Fulvetta is the lowest among these three species (Table 1). As a consequence of their adaptation to the elevation, the Spectacled Fulvetta began pairing and laying eggs about half a month earlier than the other two fulvettas. This conforms to the general pattern that birds breed earlier at lower altitudes or latitudes [23,24].

Birds of the same taxonomic group are often observed to build similar nests [25,26]. The open cup-shaped nest of the Spectacled Fulvetta was very similar to that of the White-browed Fulvetta [9] and Gray-hooded Fulvetta [10]. Other facets, including egg morphology, incubation period, and nestling period, did not show much difference between these three *Fulvetta* species, although they differ significantly in elevation, except for egg mass and clutch size (Table 1). Spectacled Fulvetta had smaller and fewer eggs, i.e., 2–3 eggs, but the clutch size could reach 4 (3 of the recorded 14 nests) in White-browed Fulvetta, and that could reach 4 (5 of the recorded 6 nests) in Gray-hooded Fulvetta as well. Since the latter two *Fulvetta* species breed at higher altitudes, the prevailing hypothesis of “smaller clutch but larger eggs in high altitude” [27,28] would predict fewer eggs of them when compared to Spectacled Fulvetta. Our findings contradict this prediction. Another case study on the Rock Sparrow (*Petronia petronia*) also violated this prediction since the Tibetan population had a larger clutch size than that of the European populations [29]. Other studies suggested that smaller clutch sizes might be a response to an increase in nest predation [30,31]. Our study site is adjacent to an urbanized area, and the overall nesting success is lower (45.45%) than that of other babbler species [32,33]. We thus hypothesize that high pressure from nest predation may be responsible for the smaller and fewer eggs.

We found that some artificial materials were used to construct the nests. The plastic filament appeared in the lining layer of nests of *F. r. sordidior* and *F. vinipectus* [9], while it was not recorded in nests of *F. cinereiceps* [10] and *F. r. ruficapilla* [13]. Since plastic items have only been widely used for decades, their use in avian nests would be an innovation in the choice of nest material and require a novel nest-building technique. It was also recorded in Chinese Bulbul (*Pycnonotus sinensis*) [34], and Common Blackbird (*Turdus merula*), whose nests with plastic were initially built earlier but subsequently suffered from higher rates of predation than those without plastic [35]. Birds use of plastic in nests indicates the degree of urbanization, and existing studies have summarized the explanatory hypotheses to explain the changes in nest materials due to urbanization, including the availability hypothesis, age hypothesis, adaptive hypothesis, and new location hypothesis [36]. The difference in nest material between the two subspecies of the Spectacled Fulvetta is more in line with the availability hypothesis, which holds that availability plays a vital role in the choice of nest materials. From the perspective of avian behavior adaptation to urbanization, it is worth exploring the plastic uses in the nests of other birds from the same area of this study in the future [36].

## 5. Conclusions

In the present study, we provide the first detailed information about the breeding biology of the Spectacled Fulvetta in Southwest China. Meanwhile, we compared its life history traits with those of its other two high-elevational counterparts in the light of life history theory. The breeding period of the Spectacled Fulvetta is from March to June. Spectacled Fulvetta build nests in lush shrubs. The clutch size is 2.73 ± 0.45 (*n* = 11), most of which are 3 eggs. Eggs were ovoid with white coloration and covered at the blunt end by dark blotches of varied size. The incubation period is 13–14 days (*n* = 3), and the nesting period is 12–14 days (*n* = 4). Both parents participate in the breeding process, with an overall breeding success rate of less than 50%. Compared with the other two *Fulvetta* species that breed at higher altitudes, the Spectacled Fulvetta starts earlier on pairing and egg-laying. In addition, Spectacled Fulvetta, which has smaller clutch sizes and smaller eggs, can use plastic as the nest material. We hypothesized that it is the consequence of adaptation to urban surroundings.

## Figures and Tables

**Table 1:** Comparison of breeding characteristics for the three species of the *Fulvetta* genus.

Species	Breeding Site	Elevation (m a.s.l.)	Breeding Period	Clutch Size	Fresh Mass (g)	Egg Length/mm	Egg Width/mm	Incubation Period (d)	Nestling Period (d)	References
*F. r. sordidior*	Yunnan Province	1918–2106	March–June	2.73 ± 0.45 (*n* = 11)	1.28 ± 0.13 (*n* = 5)	17.21 ± 0.43 (*n* = 5)	13.02 ± 0.48 (*n* = 5)	13.67 ± 0.47 (*n* = 3)	13.00 ± 0.71 (*n* = 4)	This study
*F. r. ruficapilla*	Shaanxi Province	1050–2200	April–June	4 (*n* = 2)	——	——	——	——	15	Li et al. 2013 [13]
*F. vinipectus*	Yunnan Province	2700–3600	April–May	2.64 ± 0.93 (*n* = 14)	1.47 ± 0.13 (*n* = 14)	16.7 ± 0.8 (*n* = 33)	12.8 ± 0.5 (*n* = 33)	17 (*n* = 1)	14 (*n* = 1)	Wang et al. 2016 [10]
*F. cinereiceps*	Sichuan Province	2500–2900	April–June	3–4 (*n* = 6)	1.6	17.8	13.0	15–16	12	Huang et al. 1988 [9]

## Data Availability

The data presented in this study are available upon request from the corresponding authors.
